# Erythropoietin Suppresses the Hepatic Fibrosis Caused by Thioacetamide: Role of the PI3K/Akt and TLR4 Signaling Pathways

**DOI:** 10.1155/2023/5514248

**Published:** 2023-08-22

**Authors:** Marawan A. Elbaset, Bassim M. S. A. Mohamed, Passant E. Moustafa, Dina F. Mansour, Sherif M. Afifi, Tuba Esatbeyoglu, Sahar S. M. Abdelrahman, Hany M. Fayed

**Affiliations:** ^1^Pharmacology Department, Medical Research and Clinical Studies Institute, National Research Centre, 33 El-Bohouth St., Dokki, P.O. Box 12622, Cairo, Egypt; ^2^Department of Pharmacology and Toxicology, Faculty of Pharmacy, Galala University, Attaka, Suez, Egypt; ^3^Pharmacognosy Department, Faculty of Pharmacy, University of Sadat City, Sadat City 32897, Egypt; ^4^Department of Food Development and Food Quality, Institute of Food Science and Human Nutrition, Gottfried Wilhelm Leibniz University Hannover, Am Kleinen Felde 30, Hannover 30167, Germany; ^5^Department of Pathology, College of Veterinary Medicine, Cairo University, P.O. Box 12211, Cairo, Egypt

## Abstract

Erythropoietin (EPO) is recognized for its function in erythropoiesis; however, its potential antifibrotic effect against liver fibrosis remains unknown. This study examined whether EPO affects thioacetamide (TAA)-induced liver fibrosis by concentrating on the Toll-like receptor 4 (TLR4) cascade and the phosphatidylinositol 3-kinase (PI3K)/Akt pathway as possible pathways. Male Wistar rats were randomized into four groups, which included: the negative control group, the TAA group (intraperitoneal; TAA 100 mg/kg three times per week for 2 weeks), and EPO-treated groups (150 and 300 IU/kg, i.p.) for 2 weeks after TAA injections. EPO attenuated hepatic fibrosis in a dosage-dependent way, as manifested by the diminution in serum alanine aminotransferase and aspartate aminotransferase activities, as well as the increase in albumin level. EPO inhibited the increase in tissue levels of tumor necrosis factors-*α*, interleukin-1*β*, transforming growth factor-*β*1, and TLR4 and raised tissue levels of PI3K and *p*-PI3K. EPO antioxidant properties were demonstrated by restoring hepatic glutathione and superoxide dismutase by preventing the accumulation of hepatic malondialdehyde. Further, EPO increased the protein expression of PI3K and Akt and decreased TLR4 protein expression. Immunohistochemically, EPO treatment altered tissue histology and downregulated mitogen-activated protein kinase protein expression. Overall, the research suggested that EPO could prevent TAA-induced hepatic fibrosis through upregulating the PI3K/Akt signaling cascade and downregulation the TLR4 downstream axis.

## 1. Introduction

A harmful result of the healing process associated with persistent liver damage is liver fibrosis, caused by various reasons, including hepatitis B virus, hepatitis C virus, excessive alcohol consumption, and nonalcoholic steatohepatitis. One of the leading causes of morbidity and mortality globally is liver cirrhosis, which develops from progressive liver fibrosis [1–3]. Hepatic stellate cells (HSCs) are vital to the fibrogenic process. Following a fibrogenic stimulation, HSCs transform from dormant “vitamin A-storing cells” to triggered “myofibroblast-like cells.” Additionally, fibrotic mediators and transcription factors are produced by activated HSCs in both paracrine and autocrine ways to sustain and accelerate the fibrotic process [4, 5]. Although significant efforts have been made to halt the fibrogenesis process by blocking key pathways, yet there is no therapy approved by the US Food and Drug Administration [6].

Phosphatidylinositol 3-kinase (PI3K) is a crucial signaling molecule in the liver that regulates a various cellular processes, including migration, adhesion, proliferation, and survival [7]. Additionally, it has been noted that the PI3K/Akt pathway plays a crucial role in inhibiting the cell apoptosis caused by several stimuli, and accumulated experimental data revealed a cross-talk among PI3K/Akt initiation and Nrf2/HO-1 gene activity [8, 9]. The PI3K/Akt pathway controls the generation of hepatocytes through mTOR, a downstream molecule, and it also possesses antiapoptotic and antioxidant properties [10]. By activating the G protein-coupled receptors, liver damage with succeeding discharge of proinflammatory cytokines like tumor necrosis factors (TNF)-*α* stimulates PI3K/Akt signaling. This pathway's activation counteracts harmful conditions via diverse mechanisms, including antioxidant and antiapoptotic characteristics [11, 12]. Moreover, increasing experimental data suggests that PI3K/Akt activation increases the expression of the HO-1 gene and that the beneficial effects of HO-1 may be closely related to the protective benefits of this signaling cascade [13].

The Toll-like receptors (TLRs) are a family of highly conserved receptors that help the host to detect microbial infection and recognize pathogens. TLR4 may be related to inflammation and fibrosis; according to recent investigations [14], TLR4 has two transduction pathways: “MyD88-dependent and non-MyD88-dependent” [15]. Through these pathways, TLR4 activates the liver's nuclear factor kappa B (NF-*κ*B), which causes the generation of proinflammatory cytokines [16, 17]. According to earlier research, high levels of TLR4 lead to the formation of inflammatory and fibrotic changes [18]. It has been shown in animal models of chronic liver damage that blocking the TLR4 signaling by modifying Lipopolysaccharide synthesis diminished liver damage [19]. Therefore, reducing TLR-4 expression may prevent fibrosis of the liver.

Through attaching to its particular binding site on erythroid progenitor cells, the glycoprotein hormone erythropoietin (EPO), which is produced in the fetal liver and kidney, causes erythropoiesis. The brain, kidney, liver, pancreatic islets, and endothelial cells are among the non-erythroid tissues and cells expressing EPO receptors [20]. It is previously reported that after subcutaneous (SC) treatment, EPO reaches its peak plasma concentrations 12–18 hr later, with a bioavailability of 30%. Peak plasma concentrations following SC injection are about 20% of those seen after IV administration. However, when comparing SC to IV administration, the slower absorption results in a 30% reduction in medication dosage [21]. EPO promotes organ regeneration and reduces fibrosis because it is a multipurpose molecule, and many different organs express their receptors [22]. EPO significantly reduced inflammation and hepatic injury in a tension-overstress rat experiment exposed to aorta constriction in the abdomen area [23]. Additionally, it has been suggested that EPO may be a robust cardioprotective drug and a probable ingredient in anti-fibrotic therapy [24]. The protective mechanism may involve preventing the release of inflammatory mediators such as the interleukin (IL) family, TNF, and transforming growth factor (TGF-*β*) [25]. EPO binding to EPORs activates several signaling cascades, including PI3K/Akt. Through the activation of PI3K and the subsequent phosphorylation of its downstream Akt to limit the inflammatory cascade events, EPO treatment reduced renal ischemia-reperfusion injury [26]. EPO prevented myocardial fibrosis by downregulating TLR4 expression and activating the PI3K/Akt [27]. For this purpose, our goal was to investigate the potential therapeutic value of EPO against TAA-induced hepatic fibrosis by concentrating on TLR4 and PI3K/Akt signaling pathway modification.

## 2. Materials and Methods

### 2.1. Animals

Twenty-four adult male Wistar rats (6–8 weeks; 180–220 g) were obtained from the “Animal House Colony at the National Research Centre (NRC, Egypt).” Rats were housed in a 12 hr light/12 hr dark cycle at ambient temperature (25°C).

### 2.2. Chemicals

Thioacetamide (TAA) was obtained from “Sigma–Aldrich Co. (St Louis, MO, USA).” EPO was acquired from “Eprex®, Janssen-Cialg, Schaffhausen, Switzerland.”

### 2.3. Experimental Design

**Figure 1:**
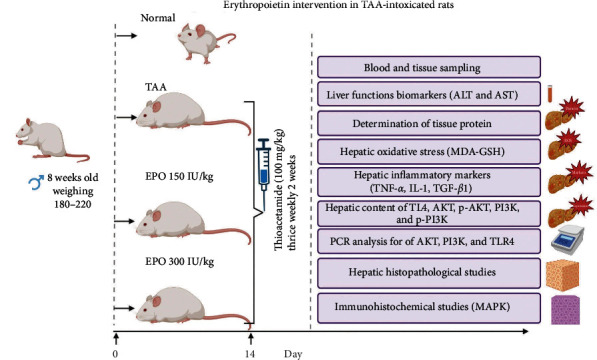
Experimental design.

Rats had a 1 week acclimation period before being randomly divided into four groups, each consisting of six animals: Rats in Group 1 (normal control group) were given an intraperitoneal (ip) injection of saline thrice weekly for 2 consecutive weeks. Rats in Group 2 (TAA group) received an ip “injection of TAA (100 mg/kg) trice weekly for 2 consecutive weeks” [28]. The TAA, we have already published previous researches with the same dose and protocol for inducing liver injury [28–30]. Rats in groups 3 and 4 were given ip injections of EPO (150 and 300 IU/kg) [31] every day for “2 weeks following TAA injection,” as shown in Figure 1. The dose was chosen as per previous research by Cetin et al. [31] investigated the solely renal function and antioxidant parameters to determine the therapeutic efficacy of EPO 150 IU/kg, i.p., on vancomycin-induced nephrotoxicity in a rat model. The literature informed our study's design, and we sought to learn more about the molecular mechanism behind TAA-induced kidney impairment at both the same dosage and its double.

### 2.4. Blood and Tissue Sampling

Blood samples were obtained 2 weeks after EPO treatment while being mildly sedated with 50 mg/kg ketamine from each rat's tail vein for obtaining serum. For biochemical testing, serum samples were kept at −20°C for analysis. Rats underwent cervical dislocation after blood samples were taken, and the livers were removed, cooled in ice water, and blotted dry. A weighted portion of each rat's liver's left lobe was removed and stored in 10% buffered neutral formalin for histopathological and immunohistochemical examinations, and another weighted portion was frozen at −80°C for molecular and biochemical research.

### 2.5. Determination of Tissue Protein

The hepatic protein content of tissue was determined as per the guidelines of the protein estimation kit (Bangalore Genei, Bangalore, India; Cat# 2624800021730).

### 2.6. Assessment of Liver Functions Biomarkers

Colorimetric analysis was performed on serum “alanine aminotransferase (ALT) and aspartate aminotransferase (AST)” activities, in addition, serum albumin levels using “Bio-diagnostic® kits, Cairo, Egypt; Cat# AS 10 61 and AL 10 31.”

### 2.7. Indicators of Oxidative Stress

Malondialdehyde (MDA) level, reduced glutathione (GSH) content, and superoxide dismutase (SOD) activity were colorimetrically assessed in liver tissue homogenate using “Bio-diagnostic® kits, Cairo, Egypt; Cat# MD 25 29, GR 25 11 and SD 25 21.”

### 2.8. Profibrotic and Inflammatory Indicators in the Liver Using an ELISA

According to the manufacturing guidelines of “Sunlong Biotec Co. LTD, Zhejiang, China,” TNF-*α*, IL-1*β*, and TGF-*β*1 were evaluated in liver homogenates (Cat# SL1761Hu, SL0402Ra, and SL1423Ra).

### 2.9. Hepatic Content of TL4, AKT, p-AKT, PI3K, and p-PI3K

According to “MyBioSource's, San Diego, USA instructions,” PI3K, *p*-PI3K, AKT, *p*-AKT, and TLR4 were evaluated in liver homogenates (Cat# MBS260381, MBS702819, MBS3807575, MBS9511022, and MBS705488).

### 2.10. QRT-PCR Analysis for AKT, PI3K, and TLR4 Expression in the Liver Tissues RNA Extraction

Direct-zol RNA Miniprep Plus “Cat# R2072,” ZYMO RESEARCH CORP (California, USA; “Cat# R2072”) was used to extract total RNA from homogenized tissues from all four groups.

### 2.11. Real-Time PCR

For reverse transcription and PCR of the extracted RNA, Thermo Fisher Scientific (Waltham, Massachusetts, USA) provided the SuperScript IV One-Step RT-PCR kit (Cat# 12594100). A thermal profile was conducted using a 96-well plate StepOne equipment (Applied Biosystems, Massachusetts, USA) as follows: Reverse transcription takes 10 min at 45°C, RT inactivation takes 2 min at 98°C, and the initial denaturation phase requires 40 cycles of 10 s at 98°C, 10 s at 55°C, and 30 s at 72°C for the amplification step. Cycle threshold (Ct) was used to express the data following the RT-PCR run for the target genes and housekeeping genes.  Table 1 presents the oligonucleotide sequences for the forward and reversed primers. “Normalization for variation in the expression of target genes AKT, PI3K, and TLR4 was performed referring to the mean critical threshold expression values of GAPDH housekeeping gene by the *ΔΔ*Ct method, and the relative quantitation of each target gene was quantified according to the calculation of 2^−*ΔΔ*Ct^ method.” Table 1 presents the oligonucleotide sequences for the forward and reverse primers.

### 2.12. Histopathological Examination

Rats from various groups were uniformly processed into “paraffin slices after a 24 hr fixation in 10% buffered neutral formalin.” The samples were cleaned in “distilled water, dehydrated in ethanol dilutes, clarified in xylene.” Finally, paraffin blocks were prepared and chopped into 4–5 *μ*m thick portions. After mounting the tissue slices on glass slides, they were deparaffinized and stained with “hematoxylin and eosin (H&E)” [32]. A trained investigator blinded for the duration of sample identification to avoid bias conducted all histopathology studies.

### 2.13. Immunohistochemical Examination

The other paraffin section from each group was used for immunohistochemical detection of the expression of mitogen-activated protein kinase (MAPK) in various experimental groups using avidin–biotin-peroxidase according to the method described. For the purpose of detecting antigen–antibody complexes, liver slices were treated with monoclonal antibodies for MAPK (Abcam, Cambridge, MA, USA) at a dilution of 1 : 200 (v/v) and Vactastain ABC peroxidase kit (Vector Laboratories, New Jersey, USA). Chromagen 3,3-diaminobenzidine tetrahydrochloride was used to visualize each marker's expression DAB (Sigma–Aldrich, St. Louis, MO, USA). The brown staining of each marker that represents its expression was estimated using image analysis software Image J, 1.46a, NIH (Maryland, USA) and seven high-power microscopic fields.

### 2.14. Statistical Analysis

Values were assured for normality using the Shapiro test. The outcomes are represented as means ± S.E. Data were processed by one-way analysis of variance followed by the Tukey–Kramer Post hoc test. GraphPad Prism software (version 9, California, USA) was used to conduct the statistical analysis and create the figures. The significance level was set to “*p* < 0.05” for all statistical tests.

## 3. Results

### 3.1. Influence of EPO on the Serum Hepatic Function Enzymes in Rats Receiving TAA

**Figure 2:**
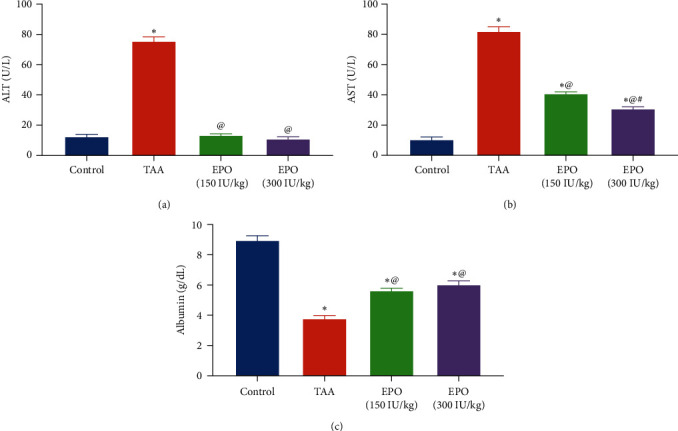
Influence of erythropoietin on the serum hepatic function enzymes in rats receiving TAA. (a) Serum ALT (U/L), (b) serum AST (U/L), and (c) serum albumin (g/dL). Each bar represents the mean ± SE of six rats. ^ ^*∗*^^vs. normal control group, ^@^vs. TAA group, ^#^vs. EPO (150 mg/kg) at *p* < 0.05. EPO, erythropoietin; TAA, thioacetamide; ALT, alanine transaminase; AST, aspartate transaminase.

TAA injection appreciably amplified serum AST and ALT activities by about 7.7 and 6-fold, whereas serum albumin level was markedly reduced by about 58% compared to the control rats. In the EPO (150 or 300 IU/kg)-treated groups, serum AST was appreciably abridged by 50% and 62%. Similarly, the ALT activity was reduced by 82% and 85% compared to the TAA group, whereas serum albumin levels were significantly elevated by 48% and 59%, as shown in Figure 2.

### 3.2. Influence of EPO on the Liver Contents of Oxidative Stress Markers in TAA-Administrated Rats

**Figure 3:**
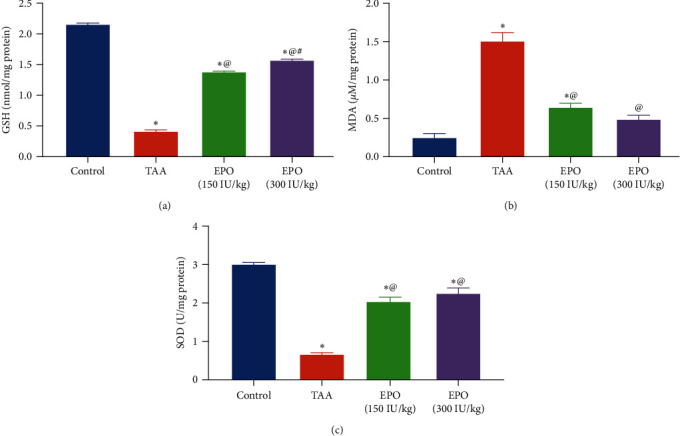
Influence of erythropoietin on liver contents of oxidative stress markers in TAA administrated rats. (a) GSH (nmol/mg protein), (b) MDA (*µ*M/mg protein), (c) SOD (U/mg protein) activity. Each bar represents the mean ± SE of six rats.  ^*∗*^vs. normal control group, ^@^vs. TAA group, ^#^vs. EPO (150 mg/kg) at *p* < 0.05. EPO, erythropoietin; TAA, thioacetamide; GSH, reduced glutathione; MDA, malondialdehyde; SOD, superoxide dismutase.

Hepatic MDA, GSH contents, and SOD activity were measured to investigate how EPO affects the liver oxidative damage caused by TAA. Injection of TAA induced a marked depletion in GSH content by 80% and SOD activity by 77% as well as a marked MDA level elevation by sixfold, compared to the negative control one. EPO (300 IU/kg) administration significantly reduced the liver MDA content by 67%. Likewise, EPO (150 or 300 IU/kg) replenished the GSH content by 3.3- and 3.7-fold and the SOD activity by 3- and 3.3-fold (Figure 3).

### 3.3. Influence of EPO on the Liver Content of Profibrotic and Inflammatory Markers in TAA-Administrated Rats

**Figure 4:**
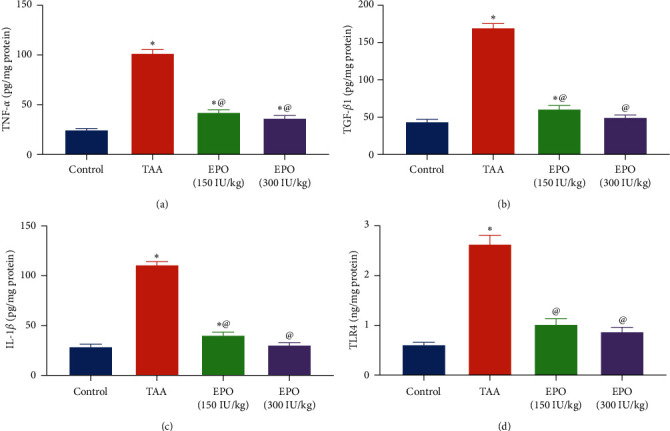
Influence of erythropoietin on the liver content of profibrotic and inflammatory markers in TAA administrated rats. (a) TNF-*α* (pg/mg protein), (b) TGF-*β*1 (pg/mg protein), (c) IL-1*β* (pg/mg protein), (d) TLR4 (ng/mg protein). Each bar represents the mean ± SE of six rats.  ^*∗*^vs. normal control group, ^@^vs. TAA group, ^#^vs. EPO (150 mg/kg) at *p* < 0.05. EPO, erythropoietin; TAA, thioacetamide; TNF-*α*, tumor necrosis factor alpha; TGF*β*1, transforming growth factor beta 1; IL-1*β*, interleukin 1 beta; TLR4, toll-like receptor 4.

TAA significantly increased TNF-*α*, IL-1*β*, and TGF-*β*1 content in the livers of rats by about fourfold compared to the normal control group (Figure 4). In comparison to the TAA-treated group, treatment with EPO (150 or 300 IU/kg) substantially reduced the rat's liver contents of the TNF-*α* by 58% and 64%, IL-1*β* 63% and 72%, as well as the TGF-*β*1 by 64% and 70% in a dose-dependent manner. EPO at a dose of 300 IU/kg revealed the most desirable effects in inhibiting inflammatory response. In addition, TAA caused a substantial elevation in the contents of TLR4 by 4.3-fold compared to the control rats (Figure 4). Meanwhile, rats treated with both doses of EPO showed a marked decrease in hepatic TLR4 content by 61% and 67% as compared to the TAA group.

### 3.4. Influence of EPO on the Liver Content of PI3K, p-PI3K, AKT, and p-AKT in TAA-Administrated Rats

**Figure 5:**
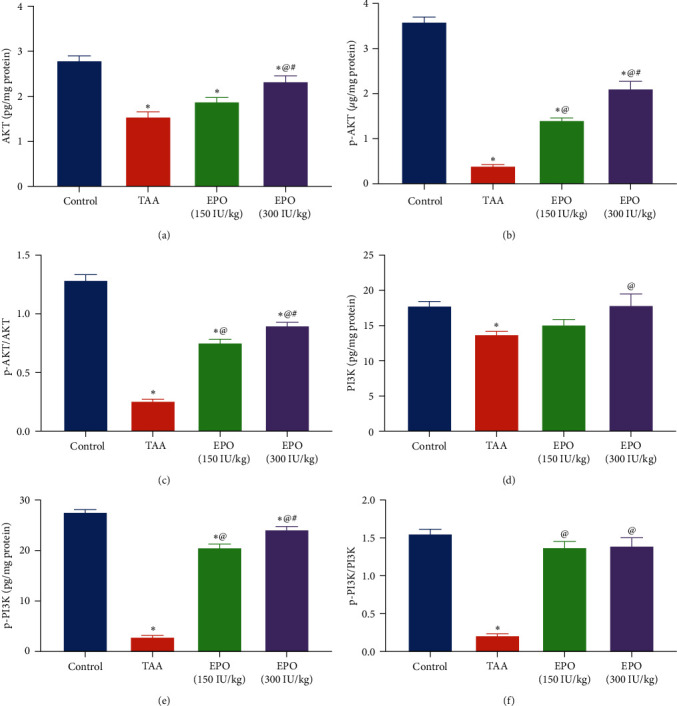
Influence of erythropoietin on the liver content of (a) AKT, (b) *p*-AKT, (c) *p*-AKT/AKT, (d) PI3K, (e) *p*-PI3K (f) *p*-PI3K/PI3K in TAA administrated rats. Each bar represents the mean ± SE of six rats.  ^*∗*^vs. normal control group, ^@^vs. TAA group, ^#^vs. EPO (150 mg/kg) at *p* < 0.05. EPO, erythropoietin; TAA, thioacetamide; PI3K, phosphoinositide 3-kinase; AKT, alpha serine/threonine protein kinase 1; *p*, phosphorylated.

There was a marked decline in hepatic PI3K and AKT levels in rats with liver fibrosis induced by TAA by 22% and 44.6% (Figure 5), and the phosphorylated of p-PI3K and p-AKT by about 89% (Figure 5) vs. the normal rats. Meanwhile, rats treated with both doses of EPO (300 IU/kg) showed a noticeable rise in the hepatic content of PI3K (1.3-fold), *p*-PI3K (8-fold), AKT (1.5-fold), *p*-AKT (5.3-fold) as well as *p*-PI3K/ PI3K and *p*-AKT/AKT ratios by 6.5 and 3.5-fold as compared to TAA group, respectively.

### 3.5. Influence of EPO on Gene Expression of AKT, PI3K, and TLR4 in TAA-Administrated Rats

**Figure 6:**
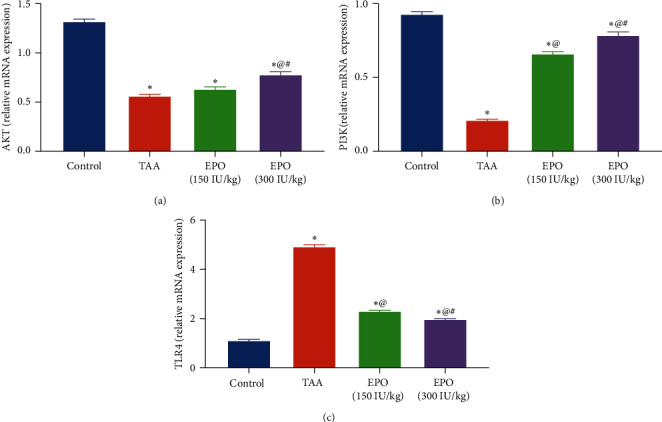
Influence of erythropoietin on gene expression of (a) *AKT*, (b) *PI3K*, and (c) *TLR4* in TAA administrated rats. Each bar represents the mean ± SE of six rats.  ^*∗*^vs. normal control group, ^@^vs. TAA group, ^#^vs. EPO (150 mg/kg) at *p* < 0.05. EPO, erythropoietin; TAA, thioacetamide; PI3K, phosphoinositide 3-kinase; AKT, alpha serine/threonine protein kinase 1; TLR4, Toll-like receptor 4.

The TAA group exhibited a substantial hepatic diminution in the protein expression of the *AKT* gene (Figure 6) and *PI3K* gene (Figure 6) by 57% and 77%, as well as a noticeable rise in the hepatic expression of the *TLR4* gene by 4.3-fold (Figure 6) vs. the normal rats. Meanwhile, EPO (150 or 300 IU/kg) treatment caused a significant increase in mRNA expression of *AKT* by 12% and 38% and *PI3K* by 3 and 3.7-fold while producing a significant decrease in *TLR4* expression, by 53% and 59% compared to the TAA group.

### 3.6. Influence of EPO on the Liver Histopathological Findings in TAA-Administrated Rats

**Figure 7:**
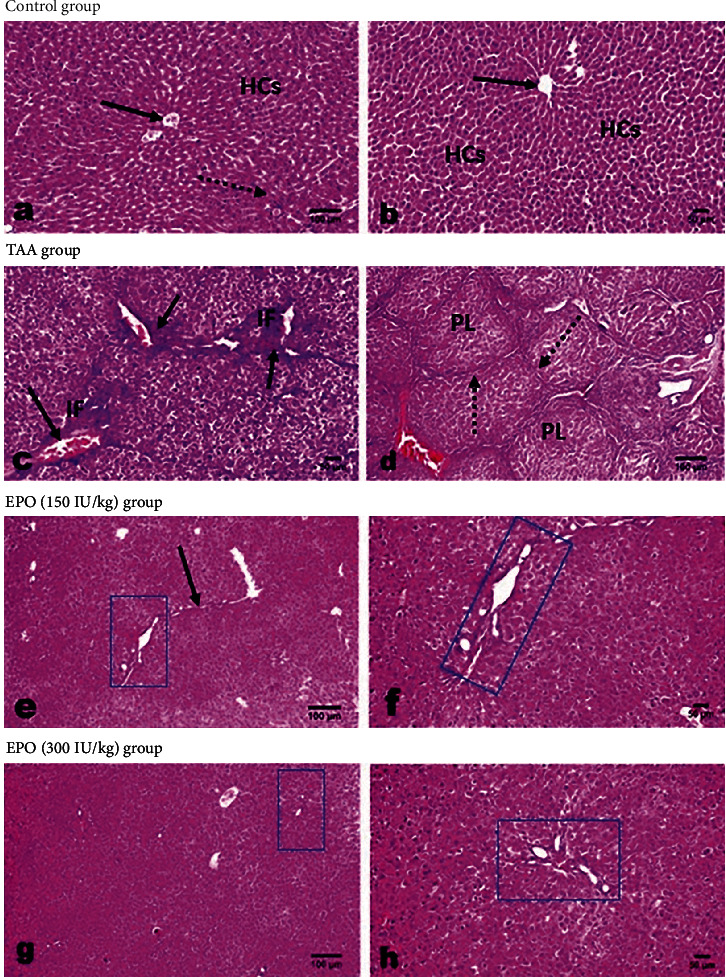
Photomicrograph of H&E-stained liver sections of various experimental groups. (a and b) The normal control group showing a normal histological structure of hepatic cells (HCs), central veins (CV), and portal areas (arrow) without any evidence of fibrous proliferation. (c and d) TAA group showing fibrous tissue proliferation (short arrow) in the portal areas with inflammatory cells infiltration (IF), congested vessels (arrow), hepatocellular nuclear changes (insert), portal to portal bridging fibrosis (dotted arrow), and parenchymal pseudo-lobulation (PL). (e–h) Erythropoietin-treated groups at low (e and f) and high (g and h) doses showing marked dose-related regression of fibrous tissue proliferation in the portal areas (rectangles), few inflammatory cells (insert) with sometimes peripheral extension (arrow) in the low dose group and mild hepatocellular degenerative changes (insert). EPO, erythropoietin; TAA, thioacetamide; low dose, 150 IU/kg; high dose, 300 IU/kg.

Liver sections of normal rats displayed regular architecture of central veins, portal areas, and cords of hepatic cells (Figures 7(a) and 7(b)). While liver sections of rats from the TAA group showed severe fibroplasia. The hepatic capsule was prominently corrugated, and normal hepatic lobulation was lost. The portal triads were severely expanded by fibrous proliferation, mononuclear inflammatory cell infiltration, cholangiolar epithelium proliferation with multiple newly formed bile ducteols, and vascular congestion (Figure 7(c)). Fibrous stands were clearly extended peripherally from one portal area toward the nearby portal area with marked bridging fibrosis and marked parenchymal pseudo-lobulation (Figure 7(d)). Within those pseudo-lobules, the hepatic cells showed vacuolar degeneration with eccentric nuclei, necrosis, and apoptosis, as well as infiltration of mononuclear inflammatory cells alongside the fibrous septa.

**Figure 8:**
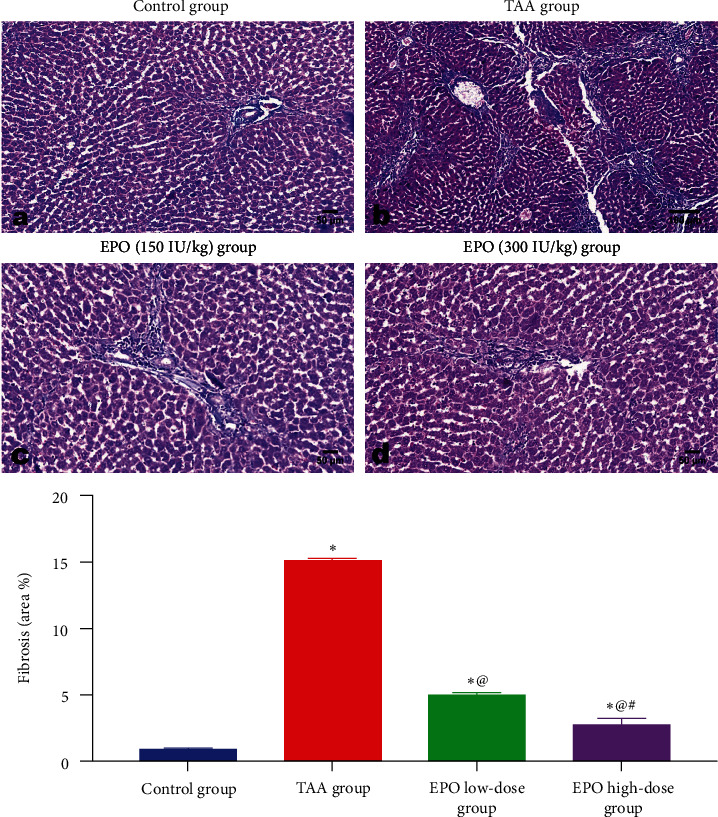
Photomicrograph of Masson's Trichrome stained liver sections. (a) Control group, (b) the TAA group, (c) the group treated with EPO at the low dose, (d) the group treated with EPO at the high dose showing the marked fibrosis and pseudolobulation of liver of TAA administrated group with its marked retraction in EPO treated groups with quantification of the fibrotic areas in liver sections of different groups presented as area percent. EPO, erythropoietin; TAA, thioacetamide; low dose, 150 IU/kg; high dose, 300 IU/kg. Each bar represents the mean ± SE of six rats.  ^*∗*^vs. normal control group, ^@^vs. TAA group, ^#^vs. EPO (150 mg/kg) at *p* < 0.05.

Regarding livers of groups treated with EPO at low (Figure 7(e)) and high (Figure 7(g)) doses, both groups showed marked dose-related regression of fibrous tissue proliferation. The low-dose group showed mild portal tract fibroplasia with sometimes peripheral extension (Figures 7(e) and 7(f)) and a few infiltrations of inflammatory cells. Hepatocellular degeneration of a moderate degree, as well as apoptosis and scattered necrosis, were observed. While livers of the high-dose group (Figures 7(g) and 7(h)) showed minimal fibrous proliferation with minimal alterations and good liver cell repair. The histo-morphometric analysis of Masson Trichrome staining presented as area percent (Figure 8) coincided with the results of H&E.

### 3.7. Immunohistochemistry Analysis

**Figure 9:**
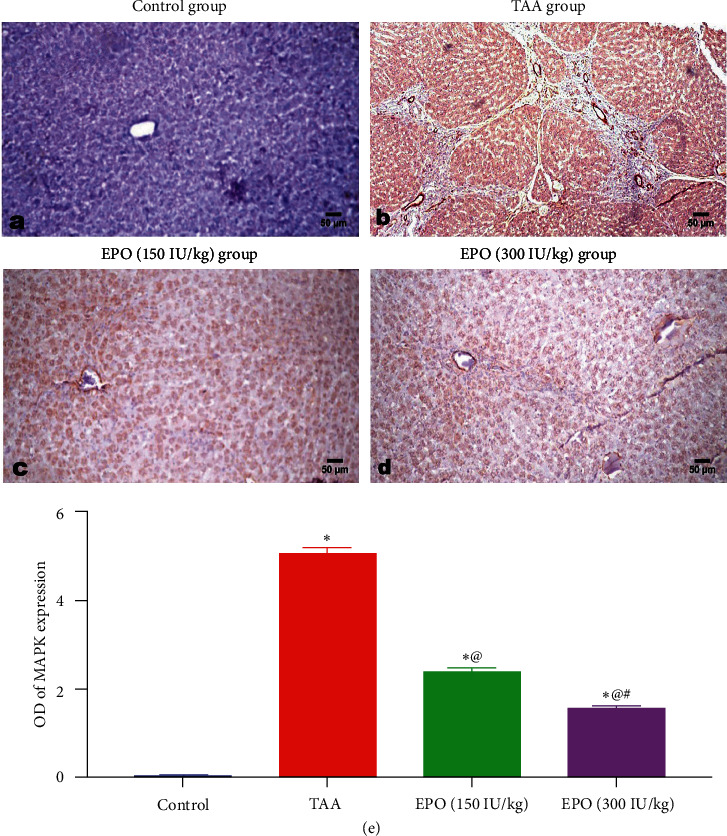
Photomicrograph of immune-stained liver sections. (a) Control group, (b) TAA group, (c) group treated with EPO at the low dose, (d) group treated with EPO at the high dose, (e) MAPK area showing marked expression of MAPK in hepatic cells in the liver of TAA administrated group and a dose-related decreased expression in EPO treated groups. Data are presented as mean ± SE.  ^*∗*^vs. normal control group, ^@^vs. TAA group, ^#^vs. EPO (150 IU/kg) at *p* < 0.05. EPO, erythropoietin; TAA, thioacetamide; MAPK, mitogen-activated protein kinase. Each bar represents the mean ± SE of six rats.  ^*∗*^vs. normal control group, ^@^vs. TAA group, ^#^vs. EPO (150 mg/kg) at *p* < 0.05.

Negative MAPK expression was visible in the livers of control rats (Figure 9). However, MAPK was found to be highly expressed in the hepatic cells of the rats whose livers had received TAA. While significantly diminished MAPK immuno-expression was seen in groups treated with EPO, particularly after high-dose administration. Compared to the other treated groups, the TAA-administered group's MAPK expression was significantly elevated.

## 4. Discussion

We demonstrated that EPO exerts significant protection for the liver against tissue injury. Our results are in harmony with previous studies on different tissues [33–35]. Indeed, we provide strong evidence that EPO has a potent tissue-protective effect on liver tissue. Several key observations were demonstrated in our study. First, we showed that EPO treatment attenuated TAA-induced liver injury by a process involving the normalization of TLR4 signaling. Second, the hepatoprotective effect was associated with an enhanced PI3k/AKT signaling pathway.

As the detailed molecular pathways mediating the beneficial effects of EPO in TAA-induced liver damage have never been demonstrated, we studied the hepatoprotective impact on several crucial parameters, including the main pathways of antioxidant and inflammatory processes.

We investigated and analyzed hepatic cell integrity as a major parameter in liver injury. In line with the histopathological findings, EPO reduced the liver's enzymes, which were expressively augmented in the animals intoxicated with TAA, suggesting enormous hepatic impediments after intoxication to the hepatotoxin and rescue of that effect by EPO. The liver function restoration by EPO was also demonstrated by the increase of albumin levels and liver enzymes compared to the low levels in the TAA group.

The antioxidant defense system is a critical component for cell protection. Oxidative stress, a major effect of TAA hepatotoxin, is hindered by antioxidant enzymes such as “SOD, catalase (CAT), and glutathione peroxidase, as well as nonenzymatic electron receptors such as GSH” [36, 37]. GSH plays a particular role in the cellular detoxification system and collaborates with GST to remove toxic metabolites. Indeed, GSH is reported as a potential measure of the cell's antioxidant ability [38, 39]. In the current research, GSH levels were decreased in the TAA-treated group, and EPO administration was capable of rescuing its ablation by TAA administration.

A byproduct of polyunsaturated fatty acid peroxidation in cells is MDA. The overproduction of MDA is caused by an increase in free radicals [40]. As predicted, the MDA levels in TAA were increased. Nevertheless, EPO treatment restored MDA levels to comparable values with the control group. These data infer that EPO can hinder oxidative stress and restore normal antioxidant balance. It is worth mentioning that EPO exerts antioxidant activity directly or indirectly. The direct scavenging action is achieved by the molecular sugar moiety. On the other hand, the indirect action is through the activation of antioxidant effectors enzymes such as SOD and CAT [33, 41–43]. Furthermore, EPO antioxidant activity was shown to involve the induction of Bcl-2 [34].

Inflammation is a major factor in many hepatic disorders. The inflammatory progression causes parenchymal injury, which can lead to hepatic scars and hepatocellular carcinoma [9]. The close association between inflammation and the generation of reactive oxygen species (ROS) has been established in a variety of disease states, including those affecting the hepatic cells. TLRs, a collection of receptors, could be perceived as a link between innate immunity and inflammatory process. TLR4 is investigated in different pathologies and found to play a role in hepatic injury resulting from infection or toxins [44]. TLR4 controls downstream pathways that control the expression of proinflammatory genes and cytokines involved in cell death and survival [45]. Further, ROS plays a critical role in the activation of NF-*κ*B, which subsequently activates the effector inflammatory cytokines [46]. Upon activation of the TLR4 signal pathway, the NF-*κ*B p65 translocates to the nucleus, inducing the transcription of inflammatory cytokines like TNF-*α* [47]. TNF-*α* is secreted by Kupffer cells and acts as a central mediator of inflammation [48]. Further, IL-1*β* is released from activated macrophages and has a vital role in hepatocyte necrosis [49]. TLR4 expression was found to be exceedingly elevated in the TAA group compared to the control rats in this study. Likewise, findings were described in a similar experiment investigating the role of TLR4 signaling in a TAA-induced hepatic liver injury and fibrosis model [50]. EPO was able to diminish the increase of TLR4. This led us to investigate the downstream inflammatory cytokines. It is a usual finding in liver injury induced by TAA to detect an upsurge in pro-inflammatory cytokine content in experimental animals [51]. In the current study, TLR4 downstream mediators, the pro-inflammatory cytokines IL-1*β* and TNF-*α* levels increased in TAA-administrated animals. EPO corrected the inflammatory status, as evidenced by a reduction in IL-1*β* and TNF-*α* levels in liver tissue after treatment. These results indicate EPO efficacy in blunting the inflammatory process at downstream of the TLR4 signaling pathway.

The control of cell survival and death is greatly influenced by the PI3K/AKT signaling cascade, an essential intracellular signal transduction system. The action of AKT that promotes survival is principally regulated by two mechanisms. First, AKT endorses cell vitality by directly phosphorylating transcription factors, resulting in the negative regulation of death-promoting genes or the positive regulation of prosurvival genes [52–54]. Second, AKT promotes cell survival by directly phosphorylating the apoptosis cascade's primary regulators. When AKT is activated, it phosphorylates the Bcl-2-associated death promoter (BAD), causing BAD to dissociate from “Bcl-2 and Bcl extra-large protein,” efficiently blocking BAD-triggered apoptosis [52, 54].

Several lines of evidence show that PI3K/AKT cell survival pathway is a target of EPO and is involved in the protection of tissues against chemical and physical insults. For instance, kinase inhibitors targeting PI3K/AKT pathways inhibited EPO's ability to reduce neuronal apoptosis caused by hypoxic cell injury and death in cultured neurons. More, treatment with small molecule inhibitors targeting the PI3K/AKT pathways inhibited the cardio-protective effect of EPO preconditioning [30, 50, 51]. Moreover, EPO's cardio-protective effect during doxorubicin or hypoxia-induced apoptosis was dependent on PI3K-AKT pathway activity and was associated with increased GSK3 inhibition by phosphorylation. Our observations are in the same context as previous studies; we showed that the hepatoprotective effect of EPO is associated with enhanced p-PI3K/ PI3k and *p*-AKT/AKT ratios as a strong indication of involvement of PI3K/AKT in the rescue effect against tissue injury in TAA induced liver damage [55, 56].

The “p38 MAPK signaling pathway” is linked to the generation of inflammatory and profibrotic mediators [57], and it plays a concrete role in the pathogenesis of fibrosis through enhancing the synthesis of the extracellular matrix. When this pathway is activated, p38 MAPK is phosphorylated and leads to the activation of downstream kinases. Further, it activates the transcription factors involved in the generation of a biological state that enhances tissue fibrosis [58].

EPO has been shown to have a dynamic effect on MAPK activity. For example, it enhances MAPK activity during the early stages of erythropoiesis while it decreases its activity at the late stages of erythroid terminal maturation [59].

**Figure 10:**
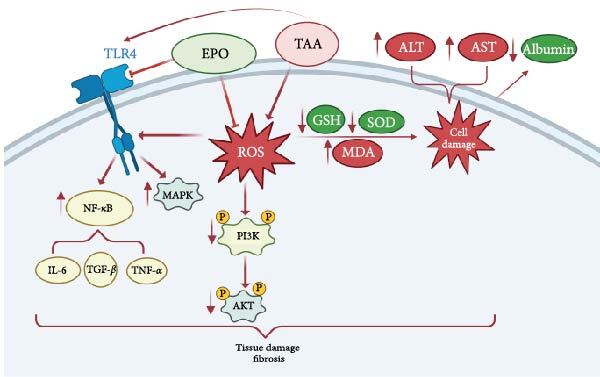
Illustration of an erythropoietin-associated pathway that suppresses the hepatic fibrosis caused by thioacetamide. EPO, erythropoietin; TAA, thioacetamide; MAPK, mitogen-activated protein kinase; ALT, alanine transaminase; AST, aspartate transaminase; GSH, reduced glutathione; MDA, malondialdehyde; SOD, superoxide dismutase; TNF-*α*, tumor necrosis factor alpha; TGF*β*1, transforming growth factor beta 1; IL-1*β*, interleukin 1 beta; TLR4, Toll-like receptor 4; PI3K, phosphoinositide 3-kinase; AKT, alpha serine/threonine protein kinase 1; *p*, phosphorylated.

In the present study, we showed that EPO treatment led to a decrease in MAPK expression in the livers treated with TAA. This demonstration was associated with less tissue damage or progress of fibrosis. The data suggest a role of MAPK in the EPO protective effect against the development of hepatic fibrosis. The illustration of the associated pathway is shown in Figure 10.

## 5. Conclusions

In conclusion, the current research reports that in the liver tissue, MAPK, TLR4, and PI3K/AKT signaling pathways are modulated in response to EPO. The phenotype we show is the downregulation of TLR4 signaling and activation of PI3K/AKT and MAPK. This led to the protection of the liver tissue from ROS, inflammation, and dysregulated repair by fibrosis. We document that these molecular events mediate the EPO effect in the inhibition of liver fibrosis and restoration of liver function.

## Figures and Tables

**Table 1:** List of primers used in qPCR.

Gene		Sequence (5′-3′)	
*AKT*	F	ATGGACTCAAACGGCAGGAG	NM_033230.2
	R	TCCTTGGCAACGATGACCTC	

*PI3K*	F	CAGGAGCGGTACAGCAAAGA	XM_017590649.2
	R	GCTGTCGATGATCTCGCTGA	

*TLR4*	F	ACAGGGCACAAGGAAGTAGC	NM_019178.2
	R	GTTCTCACTGGGCCTTAGCC	

*GAPDH*	F	ACGGGAAACCCATCACCATC	XM_039107008.1
	R	CTCGTGGTTCACACCCATCA	

## Data Availability

All relevant data are within the manuscript.
